# Experience in implementing adolescent friendly health services in rural districts of Bangladesh

**DOI:** 10.1371/journal.pgph.0003930

**Published:** 2024-11-14

**Authors:** Farhana Shams Shumi, Abu Sadat Mohammad Sayem, Nawshin Torsha, Abu Sayeed Md. Abdullah, Moonmoon Aktar, Abdul Halim, A. K. M. Fazlur Rahman

**Affiliations:** 1 United Nations Children’s Fund (UNICEF), Agargaon, Dhaka, Bangladesh; 2 Centre for Injury Prevention and Research Bangladesh (CIPRB), Mohakhali, Dhaka, Bangladesh; PLOS: Public Library of Science, UNITED STATES OF AMERICA

## Abstract

The government of Bangladesh has initiated Adolescent Friendly Health Services (AFHS) at health facilities to improve access of adolescents to quality health care. This study aimed to document the AFHS program experiences and interventions implemented in four districts of Bangladesh. The study adopted review of literature, relevant project documents, research reports and analysis of secondary data on AFHS. The secondary data was extracted from the government District Health Information System (DHIS-2) and the HMIS (Health Management Information System) of Family planning department of the four study districts in Bangladesh from 2017 to 2019. Introduction of the AFHS program in Government health facilities had a positive impact on raising awareness among the community. It increased the proportion of adolescents receiving health services (from 6% in the pre-intervention period to 86% in the post-intervention period). The involvement of school authorities in disseminating AFHS program activities resulted in a 68.51% rise in adolescent referrals from schools. Publicity of the program and privacy of the service recipients are considered as enabling factors for the acceptance of AFHS. However, training of the service providers on comprehensive adolescent healthcare packages and expanding logistics support to the health facilities are needed for improvement of the overall service. The government of Bangladesh is committed to implementing adolescent friendly services, evident by adoption of national level strategy and guidelines through government health system, using existing resources with an additional financial support from development partners. The IEC (Information, Education and Communication) materials on AFHS, developed by different organizations were used for awareness program on AFHS. The healthcare providers were oriented/trained on AFHS, mental health, nutrition and counseling skills. Multi-sectoral collaboration at all levels: national to local level (schools, communities, adolescents club) were adopted for sustainability of this initiative.

## Introduction

Adolescents make up over 1.3 billion of the global population [1] and in Bangladesh, 32 million adolescents constitute about 21 percent or one-fifth of the total population [2]. In many low and middle income countries (LMICs), adolescents face several challenges, including early marriage, high rates of fertility, poverty, lack of education, and social discrimination. More specifically, adolescent girls experience gender-based discrimination resulting in higher secondary education dropout rates, sexual abuse, domestic violence, and childbearing [3]. In Bangladesh, adolescents face a number of physical problems, like–headache, allergy, menstrual problems, malnutrition, and psychosocial problems such as- depression and anxiety among the school-going youths [4, 5]. In addition, a small proportion of the adolescent population have knowledge on reproductive health and child marriage issues [6].

A way to combat these problems is to introduce access to Sexual and Reproductive Health (SRH) information and services. SRH information and services are considered to bring a positive impact on the health and social outcomes of adolescent people across the LMICs [7]. Studies show that enforcing strict laws or regulations has little impact to reduce early marriage, an area of concern for adolescents. For example, several African countries successfully reduced early marriage by 10% in the adolescent age group, and only three countries had strong legal settings against the phenomena of early marriage (Ethiopia, Liberia, and Sierra Leone). The desired reduction in early marriage can be achieved by simultaneously increasing girls’ education and financially empowering women [8]. This renders the need to incorporate Sexual and Reproductive Health (SRH) information in the existing health system.

As an LMIC country, Bangladesh also suffers from the issue of child marriage. From the latest report, 51% of women were married before the age of 18. In addition, 24% of adolescents aged 15–19 years had their first live birth before 18 years. This age group of adolescents also sees a high fertility rate (83 births / 1000 women aged 15–49) in Bangladesh [9]. In this context, Bangladesh is stepping towards lessening the adolescent fertility rate as well as increasing adolescent health care to achieve the target under SDG 3.7.2. Studies show a growing need for SRH information and service centers among teenagers in Bangladesh, which could be provided through adolescent friendly service by adequate human resources, doctors, and associates maintaining confidentiality during the service [10, 11].

Adolescent friendly health service (AFHS) is an initiative adopted in different countries, e.g., Nepal has started AFHS since 2009/10 prioritizing sexual and reproductive health and one study shows that 67.05% of adolescent study participants utilized AFHS [12]. Another study from a Malaysian state showed there were only 35.3% adolescent friendly clinic, however, clinics providing AFHS gained more positive feedback from adolescents than that of conventional clinics [13]. Similarly, government of Bangladesh has been currently focusing on AFHS initiative and in its continuation, the Ministry of Health and Family Welfare (MoHFW) has considered adolescent health as one of the important issues in reaching the Sustainable Development Goals (SDGs) and has outlined specific inputs under the National Adolescent Health Strategy (NAHS) 2017–2030. The strategy broadly included adolescent sexual and reproductive health, nutrition, mental health, substance abuse, childhood marriage, sexually transmitted infection (STI)/ reproductive tract infection (RTI), violence and injury [14]. The MoHFW established AFHS through government health facilities, schools, institutes etc. for SRH services for adolescents [15]. Since 2016, AFHS initiative in Bangladesh is being implemented in selected health facilities with the supervision of Maternal and Child Health (MCH) Services Unit of the Directorate General of Family Planning (DGFP), Directorate General of Health Services (DGHS), with technical support from relevant national institutes, non-government stakeholders and development partners [16]. There is limited documentation of result of these initiatives as well as SRH delivery on service utilization or sexual and reproductive health outcomes, evidenced by gap of literature [17].

In this context, the overall objective of this study is to document the selected features, accounts on progress and lessons learnt from the “Adolescent Friendly Health Services (AFHS)” initiative in Bangladesh implemented from 2017 to 2019 in selected districts. The researchers listed following research questions:

- How do AFHS intervention impact the utilization of health services, specially SRH services, among adolescents at facility and community levels in Bangladesh?
- What were the situations of AFHS before the interventions, and what changes and learnings have been observed post-implementation?

## Methods

The study adopted document review and secondary data analysis approach. Document review included AFHS initiative design, extracted from the desk review and presented descriptively in the result section. It also included survey reports that were originally carried out by in relation to AFHS initiative. The secondary analysis of national level adolescent health data was carried out to understand the status and service utilization of AFHS.

### Document review

The review included a number of national level documents, e.g., operational plan, national adolescent health strategy. The review also included two reports: a baseline survey and another implementation research (IR) conducted in the same study districts. Firstly, the review of the baseline survey explored adolescent health status and services in four selected districts of Bangladesh (Tangail, Gazipur, Khulna, and Jamalpur). Secondly, review of the implementation research (IR) report found out progress and outcome of the AFHS intervention. The data was compared with the baseline to depict the progress. Lessons learned were extracted from reviewing the reports. Table 1 enlists list of key documents reviewed followed by short methodology followed in above-mentioned baseline survey and implementation research (IR) (S1 Text).

**Table 1:** List of reviewed relevant documents on AFHS.

Serial No.	Name of the document / report	Description of the document	Objective of the desk review	Key findings
1.	National Adolescent Health Strategy (NAHS) 2017–2030 (Bangladesh) [14]	A national policy document developed by govt. in partnership with experts, professionals and development partners and endorsed	To identify the national policy status and strategic directions for improving the health of the adolescent population	Policy environment of AFHS
2.	Operational Plan of DGHS and DGFP [18]	It is part of the National Health, Population and Nutrition program 2017–2022, and under implementation by the government	To identify the key activities for implementing the NAHS which also detailed activities for adolescents	Objectives, responsibilities in line with the goal contained within the strategic plan
3.	A Baseline Survey of AFHS: Household Survey July to August 2017 [19]	A survey report which was conducted to determine the program parameters for AFHS in the four districts of Bangladesh: Tangail, Gazipur, Khulna and Jamalpur	To explore status and utilization of adolescent health services before the implementation of AFHS in four study districts	Adolescent health problems, access to and utilization of health services: adolescent boys, girls and their mothers’ perspectives
4.	A Baseline Survey of AFHS: Health Facility Survey August to September, 2017 [20]	A survey report which was conducted to explore availability and readiness of the health facilities to provide services to the adolescents in same four districts	To explore health services provision for adolescents in government health facilities before AFHS program	Hospital readiness for AFHS in 56 public health facilities of 4 districts (Khulna, Jamalpur, Tangail and Gazipur)
5.	A Baseline Survey of AFHS: Qualitative Study Report August to September, 2017 [21]	A qualitative study report which was conducted to identify challenges and scopes of improvement for adolescent health services in Bangladesh	To understand an overview of the AFHS services, its utilization, challenges and scopes of improvement for AFHS	Stakeholders’ perception and recommendations about AFHS regarding its use, challenges and future steps: Service managers and gatekeepers
6.	Implementation Research (IR) report on AFHS October 2018 to December 2019 [22]	A study report which was conducted to explore the AFHS status for enhancing the facility-based AFHS within the existing government health system	To identify enabling and hindering factors for AFHS after intervention	Socio-economic barriers and solutions: Health care providers, school authorities, adolescents taking service and their parents

#### Methods adopted in baseline survey

The baseline survey was conducted from July to September 2017 in the four intervention districts of Bangladesh, namely- Tangail, Gazipur, Khulna, and Jamalpur; following a mixed method approach. The survey included data from- household survey, health facility study, and qualitative information from service managers. In the household survey, 1474 unmarried adolescent girls, 1232 married girls, 1503 unmarried adolescent boys and 808 mothers were interviewed following simple and systematic sampling. In addition, 134 health service providers were interviewed in 56 selected health facility survey. Another, 24 KIIs (Key Informant Interviews) were conducted (16 KIIs with service managers and 8 KIIs with gatekeepers) during the qualitative survey.

The baseline survey produced three separate reports, which is household survey support, health facility survey report, and qualitative study report. For this study, all of the reports were thoroughly reviewed to understand the status of adolescent reproductive health, AFHS services, its utilization, adolescents’ perception regarding the AFHS and, the facility readiness in the study districts.

#### Methods adopted in implementation research (IR)

The implementation research was conducted in the same districts from October 2018 to December 2019. Selected data and information were collected by researchers using a checklist. The research was conducted in two approaches–innovation implementation and scale-up. It took place in selected AFHS health facilities at district, sub-district and union level in the same districts (as baseline). The report assessed the effectiveness of AFHS and generated evidence to enhance facility based adolescent health services.

### Secondary data analysis of AFHS extracted from DHIS-2

The current study involved an analysis of existing data obtained from ongoing adolescent health service programs in Bangladesh. Datasets were collected from the District Health Information System, version 2 website (DHIS-2), and Health Management Information System (HMIS) report of family planning department in the four districts (Tangail, Jamalpur, Khulna and Gazipur) from 2017 to 2019 (S1–S4 Data).

The collected data was aggregated by age (10–14 years, 15–19 years), sex (male, female) and marital status, reported on a monthly basis including annual summary data on health and family planning services. Data was analyzed and presented graphically using Excel 365 software. The following four indicators regarding AFHS program outputs are focused:

- adolescents received services from the facility
- adolescents referred from the educational institutes
- adolescents referred from adolescent clubs and others
- sexually active adolescents using contraceptive methods

This study conducted secondary analysis of anonymized data and obtained approval from relevant authorities.

## Results

The study adopted the approach of document reviews (existing policies and survey reports) related to AFHS and analysis of secondary data on AFHS from 2017 to 2019. Result section includes- an overview of AFHS status in Bangladesh, interventions introduced for AFHS, and AFHS situations before interventions, based on the findings from document review. Findings from baseline study report were utilized to depict situation before AFHS in the government health facilities. Afterwards, findings from the IR report and secondary data analysis were summarized in the section- “changes and learnings after interventions” and “Analysis of secondary data”. IR report was reviewed to identify the barriers and recommendations for AFHS. Secondary data collected from DHIS-2 were analyzed to portray trends in service utilization of AFHS by adolescents over the years. The findings are presented under several sub-sections.

### AFHS initiative in Bangladesh

The Ministry of Health and Family Welfare (MoHFW) has developed and adopted a National Adolescent Health Strategy (NAHS) to guide the implementation of AFHS divided by administrative levels for ensuring the health and well-being of adolescents [14]. Both DGHS and DGFP have outlaid their own annual Operational Plans (OP) and activities to achieve the targets under the NAHS. In addition, other Ministries including Local Government, Rural Development and Cooperatives; Education; Social Welfare; and Women and Children Affairs work for adolescent health in collaboration with MoHFW. Besides, many development partners, including United Nations (UN) agencies, World Health Organization (WHO), International Nongovernmental Organizations (INGOs), Non-governmental organization (NGOs), and civil society, play a vital role in addressing the health needs of adolescents by supporting government of Bangladesh (GoB) [16]. The AFHS interventions (described below) were designed by MoHFW, in technical support of WHO, UNFPA, UNICEF, Bangabandhu Sheikh Mujib Medical University (BSMMU), and other professional experts adopting WHO recommended eight global standards and guidelines for quality AFHS [23, 24]. The developed AFHS program was then implemented in four districts of Bangladesh [19–22]. Moreover, several National IEC materials for AFHS have been developed by MoHFW with the technical support of UNICEF and The Embassy of Netherlands. The IEC materials focus on adolescent sexual and reproductive health and rights, which were developed through different projects of government and non-government partners [25].

Since 2015, initiatives for provision of AFHS started in a limited scale in Bangladesh, initially by establishment of dedicated counselling room for adolescents at the government health facilities (both primary and secondary tier of health facilities). At each tier, health care providers provide listed services to married and unmarried adolescent boys and girls aged 10–19 years. Services include counselling services on several topics, e.g.- puberty, nutrition, vaccination, menstrual hygiene, early marriage, violence related information and suggestions, treatment related services, such as—reproductive tract infection (RTI) / sexually transmitted infection (STI), family planning methods (for married adolescents only) etc. Health care providers who had successfully completed an approved four-day training course on AFHS by the government during the period of intervention were taken as well-trained staff. They also received additional training in counselling skills, and adolescent mental health [19, 26].

At each tier of health facility, different staff are involved in service provision, which is summarized in Table 2.

**Table 2:** AFHS service providers in different tiers of health facilities in Bangladesh [26].

Type of health facility	Place of health facility	Service provider
District Hospital	District	Medical officer (MO) / Sub-assistant community medical officer (SACMO)
Maternal and Child Welfare Centre (MCWC)	District	Medical officer–clinic (MO-clinic) / Medical officer–maternal and child health, family planning (MO-MCH-FP) referred cases from union.
Upazila Health Complex (UHC)	Upazila (Sub-district)	Sub-assistant community medical officer (SACMO)
Union Health and Family Welfare Centre (UH&FWC)	Union (Sub-upazila)	Sub-assistant community medical officer (SACMO) / Family Welfare Visitor (FWV)

### Intervention packages (Facility and community level approach)

The following key interventions for AFHS were identified aimed towards the adolescent population from the reviewed documents (IR report):

### Community approach

#### A. Dissemination of information on AFHS in school assembly

1. A short message on available “AFHS” shared at high schools during school assemblies for publicity (two to three times a week).
2. School teachers and adolescents will share the information with other adolescents and establish a referral linkage between school and facility.

#### B. Health education on adolescent health in courtyard session

1. Message sharing in the locality about AFHS targeted to adolescents and their parents.

### Facility approach

#### A. Special Service Day for adolescents in health facilities

1. Free services for adolescents from the facility within the service hour (once in three months). The aim was to familiarize the adolescents with AFHS at facility level.
2. Capacity building of health care providers on AFHS.
3. Visible billboard to disseminate the AFHS messages.

### Other related interventions

1. Integrate religious institutions for seeking support from AFHS facilities.
2. Strengthen referral linkage from school and adolescents club to facilities.

### AFHS situation before the interventions

According to data from the baseline survey (July to September 2017), most of the adolescent boys and girls and their parents (86%) were unaware of AFHS. School surveys found that 94% of adolescents did not visit health facilities. Of those who visited facilities, only 20% were referred by the community health workers. In addition, findings of the health facility survey explored that most of the health facilities (46 out of 56) intended to provide services to adolescents. However, not all the facilities have the four basic components (guidelines, trained staff, equipment, and medicines) as per WHO guidelines for adolescent health [19–21, 23]. The survey found that guidelines for providing AFHS was available only in nearly 9% of the surveyed facilities (one MCWC, one UHC and, three UHFWCs). Trained staff on AFHS in the facilities were found in only 3% of facilities back in 2017 (2 out of 56 facilities) as the training on adolescent health had not been started properly. While being generally happy with the way service providers treated adolescents, AFHS users and others described several limitations in accessing services during qualitative interviews. According to all respondent groups, lack of publicity and awareness about AFHS, lack of gender-matched sensitive service providers etc. were identified as barriers to receiving AFHS. In addition, expectations from the mothers of adolescents were explored during the baseline to enhance the AFHS. Most of them opined that adolescents should be assisted by HCPs in the health facilities while availing services regarding STI, mental health, menstrual hygiene and addiction. Utilization of BCC materials on adolescent health was not found in all the facilities except only in one UHC [20, 21].

### Changes and learnings after interventions

The study found that the percentage of adolescents attending government health facilities increased after implementing the above-mentioned interventions. The post-intervention phase reported that almost 86% of adolescents went to a health facility to evaluate their general health issues, while 21% went for adolescent health issues. Similarly, after the intervention, adolescents’ counseling ratio increased from 4% to 11%. In addition, about 6% of school-going adolescents visited AFHS centers before intervention which increased almost ten times after intervention. The study also identified that overall referral status to the facilities was increased to 46% from schools, and 39% of adolescents were referred by the parents. A number of interventions were employed, among which notable ones were training of health care providers, raising awareness on AFHS through school and adolescent clubs. In the pre-intervention assessment, nearly 8% of adolescents got the message from schools, rising to more than 90% in the post-intervention [22].

Although there was overall improvement in post-intervention compared to baseline status, a few barriers for implementation were narrated by the responders (adolescents and their parents, healthcare providers, stakeholders) in IR report, such as–shortage of AFHS trained HCPs, inadequate gender-matched service providers in the facilities, insufficient referral linkage, lack of coordination among facilities and schools. Based on the IR findings, the responders also suggested effective approaches for establishing AFHS as well as for sustainability of the AFHS program, comprising of the key interventions stated above, involving a multi-sectoral platform for the dissemination of AFHS information within the health facility, school, parents, adolescent club, and communities [22].

### Analysis of secondary data

The analysis of the secondary dataset of Oct 2017-Dec 2019 taken from DHIS-2 and HMIS of DGFP are presented here:

#### Adolescents receiving services from health facilities

From Table 3, the data shows the increasing trend of taking services from healthcare facilities among the adolescent population. According to data by age group, the number of service recipients in the age group 15–19 years was approximately more than twice that of age group 10–14 years over the time span of 2017 to 2019.

**Table 3:** Year-wise updates of adolescent friendly health services (AFHS).

Indicators	Categories	2017	2018	2019	Total
**Total service received**		17716	63553	249046	**330315 **
**Age**	10–14 years	5628 (31.77%)	17223 (27.1%)	93964 (37.73%)	**116815 **
	15–19 years	12088 (68.23%)	46330 (72.9%)	155010 (62.27%)	**213428 **
**Sex**	Boy	3282 (18.52%)	19076 (30.01%)	75852 (30.46%)	**98210 **
	Girl	14434 (81.48%)	44477 (69.99%)	173194 (69.54%)	**232105 **
**Marital status**	Married	4695 (26.5%)	11258 (17.31%)	34184 (14.37%)	**50137 **
	Unmarried	13021 (73.5%)	53773 (82.7%)	203650 (85.63%)	**270444 **
**Referred from**	School	2369 (37.43%)	4467 (70.48%)	41283 (68.51%)	**48119 **
	Adolescent Club	3960 (62.57%)	1873 (29.54%)	18972 (31.47%)	**24805 **

Results are expressed in percentage, denominator taken is total number of service recipients of each indicator.

Based on the data presented in Table 3, in 2017 AFHS users, 18.52% were boys and 81.48% were girls (about more than four times than boys). However, the number of male service takers rose over the next few years, still the ratio of girls was twice as likely than that of boys to utilize adolescent health services and information from public facilities. Similarly, married adolescent population had an increased inflow to health facilities from the year 2017 to 2019, although they occupied a relatively smaller percentage of the total service takers than the unmarried ones.

In the beginning, the number of adolescent boys and girls referred from school was 1/3^rd^ of the total referral only. According to the baseline study, publicity on AFHS started at selected areas of study districts, but more was needed to inform all the adolescents as well as school authorities. The findings indicated that in 2017, the number of youth (both boys and girls) recommended by school staff was lower, but that this number eventually grew in 2019. The IR on AFHS found that no adolescents were referred from school to AFHS in pre-intervention period, though at post-intervention, it was 68.51% from schools. The school authority also mentioned sufficient publicity on AFHS as one of the facilitators for increasing the referral system of adolescents to the health facilities (IR study on AFHS). From 2017–2019, the number of adolescents referred from schools has progressed, where there is an upsurge from 2018 to 2019 in both the referral system (from schools and from clubs) after the initiation of AFHS interventions.

Boys were also less likely to be referred to health facilities than girls at the early stage of AFHS, but their referral increased about ten-fold over the years from clubs to utilizing services from the government health facilities. In addition, lack of awareness on adolescent health among teenagers as well as in the community was one of the substantial reasons for not referring to health facilities (Baseline report on AFHS). However, effective management and coordination among facilities, schools, and adolescent/youth clubs were identified as an essential enabling factor for encouraging adolescents to utilize AFHS (IR report on AFHS). Overall referral status increased over the intervention period, which aided in increasing the flow of adolescents into facilities.

#### Sexually active adolescents using contraceptive methods

**Fig 1:**
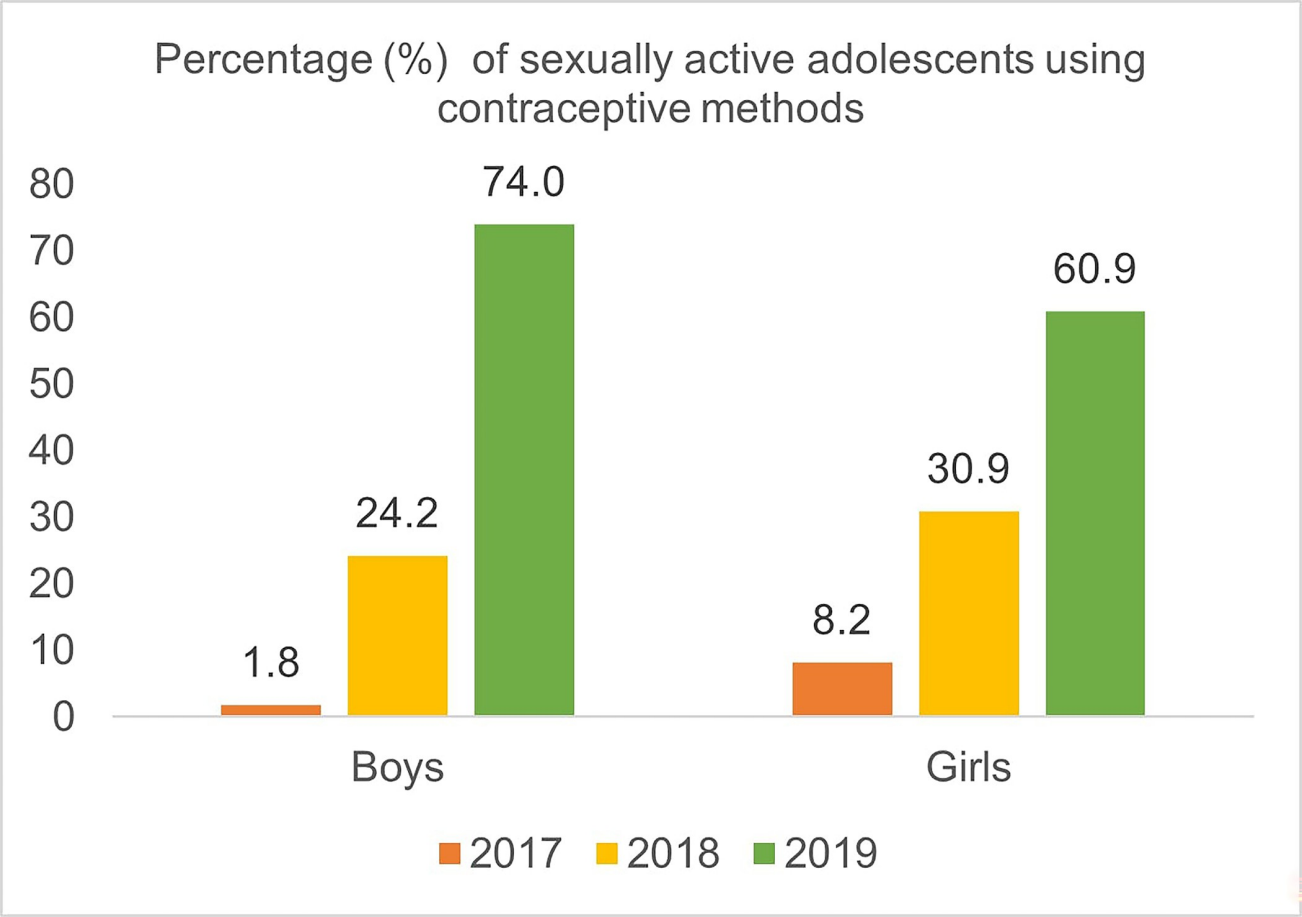
Percentage (%) of sexually active adolescents using contraceptive methods.

The data in Fig 1 shows that adolescents are more active in using contraceptive methods in recent years (2019). From the Baseline survey, a recommendation was given to facilitate female service providers for adolescent girls and male service providers for adolescent boys to ensure coziness. In addition, baseline survey and IR on AFHS reported that awareness of AFHS and privacy during receiving services were found to be effective for using contraceptive methods by adolescents. The survey assessed privacy during consultation based on three criteria: separate room, no separate room but maintains auditory privacy, and no separate room but maintains visual privacy. The study observed that merely 7% of facilities have separate rooms for adolescent health services, while 2% maintain auditory privacy and another 2% maintain visual privacy.

Overall, data analysis primarily showed the improvement of adolescent health services from the baseline survey to IR period through a sustainable intervention in government health facilities. However, detailed observation revealed major optimistic implications of adolescents’ health services along with few drawbacks in these studies and reporting systems.

## Discussion

The key findings of this study described the status of the AFHS, intervention packages of AFHS through facility and community level approach, AFHS situation before the interventions, and changes and learnings after interventions. The AFHS service with utilization of information, education and communication (IEC) materials in the community, and established referral linkage were found significantly improved after the intervention.

The pre-intervention findings in AFHS revealed that adolescents had a demand for health services, but care-seeking behavior faces obstacles due to less adolescent friendly health facilities. About 50% of service providers were trained on adolescent health in MCWC whereas, only 20% of service providers were trained in other facilities [16]. In post-intervention, capacity of health care providers increased resulting in improved service utilization at facility level. This renders the need for increased training of health care providers on AFHS. A study conducted in a Malaysian state in late 2018 demonstrated that the adolescent population were more satisfied with adolescent friendly clinic services with skilled capacity compared to services provided by conventional clinics [13].

IR identified appropriate interventions for AFHS. This IR report also shows that interventions applied in school and adolescents’ clubs have a positive influence on teachers, parents, and adolescents’ own self to receive care from health facilities. In the first round of research, it was 14.4 percent awareness about the AFHS among adolescents which was 91% in the third round. Influence of parents and peers acted as an enabling factor here [22]. Similar findings can be observed in South Nigeria, where youths were 1.81 times more likely to utilize AFHS after the implementation of interventions like service center renovation, awareness to schools and, communities and skilled service providers [27].

The IR shows that dissemination of AFHS service resulted in expansion of educational institutions referrals from 2017 to 2019 [22]. A study in Nepal has shown majority of the adolescents taking youth friendly services mostly acknowledged it from radio/television [28]. In that perspective, publicity of AFHS initiatives on mass media channels (newspapers, television) and social media sites could provide more coverage to adolescent people in general.

Adolescent boys and girls have become more likely to use contraception in recent years. The data analysis also demonstrated that assurance of privacy during AFHS services in the facilities increased the percentage of adolescents receiving contraceptive methods and SRH advice from the service providers [22]. However, one recent study in Bangladesh regarding women of reproductive age identified a number of societal obstacles faced by adolescent girls in utilizing contraceptive methods, e.g., urban-rural difference, low education level [29]. Another Bangladeshi study also discussed the issues with demand for gender-matched service provider for adolescents especially young women [10, 30]. This tendency is encouraging as it is necessary to establish age-appropriate measures for providing access to contraception among the sexually active adolescent population [31].

The interventions for AFHS designed in Bangladesh did not include any screening activities. However, one of the recommended interventions from IR was to introduce ‘Special Service Day for adolescents in health facilities’ where height-weight would be measured along with other activities [22]. It is possible to introduce a screening program along with this service day which could uplift success of the program. Literature review recommended that for a successful AFHS initiative, it would be helpful if the government established a health screening program for adolescents [32, 33].

### Limitations

This study, reviewing large datasets and evaluating facility-based adolescent health services, before and after intervention is new of its types in Bangladesh. The study worked on a pre-collected data set, some of the relevant covariates may have been overlooked by data collectors as seen in observational studies. Adolescents’ health indicators in the dataset mainly focused on health services among age-specific girls and boys. Indicators require improvement by adding mental health and gender-based violence status indicators for adolescents, as these areas have been prioritized on National Strategy for Adolescent Health 2017–2030 [14].

## Conclusion

The existing adolescent health interventions show a significant upward growth of AFHS utilization. The AFHS interventions were simple, appropriate, and sustainable for scale-up in Bangladesh. The findings and experiences gathered from this study will improve service utilization of adolescents at the utmost level. The effective coordination of different relevant organizations plays a vital role in ensuring the sustainability of AFHS initiative. The awareness of adolescents is essential to establish referral linkage between community and facility through educational institutes. The combined data can help researchers and policy makers for appropriate and contextualized action. The AFHS initiative provides opportunities to make progress towards Sustainable Development Goals in alignment with universal health coverage. In order to ensure a brighter future for upcoming generations, AFHS activities must be scaled up nationally to fulfill the healthcare needs of adolescents.

## Supporting information

S1 Text Reporting procedure. (DOCX)

S1 Data Gazipur district summary data of health and family planning 2017 to 2020. (XLSX)

S2 Data Jamalpur district summary data of health and family planning 2017 to 2020. (XLSX)

S3 Data Tangail district summary data of health and family planning 2017 to 2020. (XLSX)

S4 Data Khulna district summary data of health and family planning 2017 to 2020. (XLSX)

## References

[1] Adolescents statistics. UNICEF; 2022 [cited 2022 Aug 24]. Available from: https://data.unicef.org/topic/adolescents/overview/

[2] Report on census of the former enclaves population of Bangladesh 2017. Bangladesh Bureau of Statistics, Statistics and Informatics Division, Ministry of Planning, Government of the People’s Republic of Bangladesh; 2017. Available from: https://bbs.gov.bd/site/page/b588b454-0f88-4679-bf20-90e06dc1d10b/-

[3] Ritchwood TD, Ford H, DeCoster J, Sutton M, Lochman JE. Risky sexual behavior and substance use among adolescents: A meta-analysis. Children and Youth Services Review. 2015 May;52:74–88. doi: 10.1016/j.childyouth.2015.03.005 25825550 PMC4375751

[4] Kamrun Nahar, Koly Md., Saiful, Marc., Potenza, Rashidul Alam, Mahumud., Farzana Begum., Daniel D., Reidpath. Psychosocial health of school-going adolescents during the COVID-19 pandemic: Findings from a nationwide survey in Bangladesh. PLOS ONE, (2023). doi: 10.1371/journal.pone.0283374 36972260 PMC10042372

[5] Tahmina Banu., Tanvir Kabir, Chowdhury Tasmiah, Tahera Aziz., Nowrin Tamanna., Arni Das., Nugayer Sharmeen, et al. Health and Disease among Adolescent Urban School Girls in Bangladesh. Chattagram Maa-O-Shishu Hospital Medical College Journal, (2022). doi: 10.3329/cmoshmcj.v21i1.59752

[6] Mohammad Zahidul, Islam. High Risk Behavior and Knowledge among Female Adolescent: A Study on Rajshahi City of Bangladesh. Asian Journal of Applied Science and Engineering, (2018). doi: 10.18034/AJASE.V7I2.233

[7] Parsekar SS, Pundir P, Bevilacqua V. Reproductive, maternal, newborn, child and adolescent health and related behaviour change communication strategies in Bangladesh, Nepal and India: A narrative review. Clinical Epidemiology and Global Health. 2020 Mar;8(1):280–6. doi: 10.1016/j.cegh.2019.08.014

[8] Chandra-Mouli V, Lane C, Wong S. What does not work in adolescent sexual and Reproductive Health: A review of evidence on interventions commonly accepted as best practices. Global Health: Science and Practice. 2015 Aug 31;3(3):333–40. doi: 10.9745/GHSP-D-15-00126 26374795 PMC4570008

[9] Progotir Pathey Bangladesh—Multiple Indicator Cluster Survey 2019. UNICEF; 2019 [cited 2022 Feb 9]. Available from: https://www.unicef.org/bangladesh/media/3281/file/Bangladesh%202019%20MICS%20%20Report_English.pdf

[10] Samiha Yunus., Sabrina Sharmin., Nafisa Lira, Huq Fariha, Haseen Ali, Imam Quamrun, Nahar. Expectations of adolescents to receive reproductive health information and services from health service system: A qualitative study in Bangladesh. South East Asia Journal of Public Health, (2018). doi: 10.3329/SEAJPH.V7I2.38852

[11] Abid Hossain, l. Adolescent Health: An Unmet Demand of Time. Bangladesh Journal of Child Health, (2014). doi: 10.3329/BJCH.V38I2.21137

[12] Sharma M, Khatri B, Amatya A, Subedi N, Upadhyaya DP, Sapkota BP, et al. Utilization of adolescent friendly health services and its associated factors among higher secondary students in mid-western Himalayan mountainous district of Nepal. PLOS Global Public Health. 2023 Mar 17;3(3). doi: 10.1371/journal.pgph.0001616 36963100 PMC10022795

[13] Awang H, Ab Rahman A, Sukeri S, Hashim N, Nik Abdul Rashid NR. Adolescent-friendly health services in primary healthcare facilities in Malaysia and its correlation with adolescent satisfaction level. International Journal of Adolescence and Youth. 2019 Nov 5;25(1):551–61. doi: 10.1080/02673843.2019.1685556

[14] National-strategy-for-adolescent-health-2017-2030. MCH Services Unit, DGFP; UNICEF and UNFPA; 2016 [cited 2022 Feb 9]. Available from: https://www.unicef.org/bangladesh/sites/unicef.org.bangladesh/files/2018-10/National-Strategy-for-Adolescent-Health-2017-2030.pdf

[15] Ainul S, Ehsan I, Tanjeen T, Reichenbach L. Adolescent friendly health corners (AFHCS) in selected government health facilities in Bangladesh: An early qualitative assessment. Research Report of The Evidence Project Population Council. 2017; doi: 10.31899/rh7.1002

[16] A Baseline Survey of Adolescent Health and rights Enhancement through Innovation and System Strengthening (ADOHEARTS). MCH Services Unit, DGFP; UNICEF and UNFPA; 2017 [cited 2022 Jan 4]. Available from: https://stage-adolescent-health.dnet.org.bd/uploads/policy_guideline/1672578325_ADOHEARTS_Survey_Key_Findings.pdf

[17] Denno DM, Hoopes AJ, Chandra-Mouli V. Effective strategies to provide adolescent sexual and reproductive health services and to increase demand and community support. Journal of Adolescent Health. 2015 Jan;56(1). doi: 10.1016/j.jadohealth.2014.09.012 25528977

[18] 4th Health, Population and Nutrition Sector Programme. Health Service Division, MoHFW; 2017 [cited 2022 Mar]. Available from: http://hospitaldghs.gov.bd/wp-content/uploads/2020/01/HSM_OP_2017-22.pdf

[19] A Baseline Survey of Adolescent Health and Rights Enhancement Through Innovation and System Strengthening. UNICEF Bangladesh and The Embassy of The Kingdom of the Netherlands; 2018 [cited 2022 Mar 4]. Available from: https://stage-adolescent-health.dnet.org.bd/uploads/policy_guideline/1672578224_Baseline_ADOHEARTS_House_Hold_Survey_Report.pdf

[20] A Baseline Survey of Adolescent Health and Rights Enhancement Through Innovation and System Strengthening Health Facility Survey and Rights Enhancement Through Innovation and System Strengthening. UNICEF Bangladesh and The Embassy of The Kingdom of the Netherlands; 2018 [cited 2022 Mar 4]. Available from: https://stage-adolescent-health.dnet.org.bd/uploads/policy_guideline/1672578184_Baseline_ADOHEARTS_Health_Facility_Survey_Report.pdf

[21] A Baseline Survey of Adolescent Health and Rights Enhancement Through Innovation and System Strengthening QUALITATIVE STUDY REPORT. UNICEF Bangladesh and The Embassy of The Kingdom of the Netherlands; 2018 [cited 2022 Mar 4]. Available from: https://stage-adolescent-health.dnet.org.bd/en/survey-conference

[22] Implementation Research to enhance facility-based gender-responsive adolescent-friendly health services in selected districts of Bangladesh. UNICEF Bangladesh and The Embassy of The Kingdom of the Netherlands; 2019 [cited 2022 Mar 4]. Available from: https://stage-adolescent-health.dnet.org.bd/uploads/policy_guideline/1672578018_Final_Report_of_Implementation_Research_on_AFHS.pdf

[23] Making Health Services Adolescent Friendly. World Health Organization; 2013 [cited 2024 Feb 28]. Available from: https://www.who.int/publications/i/item/9789241503594

[24] National accreditation guideline for adolescent friendly health services. MCH Services Unit, Directorate General of Family Planning, Ministry of Health and Family Welfare; 2019 [cited 2024 Feb 12]. Available from: https://www.share-netbangladesh.org/wp-content/uploads/2021/03/Accreditation-Guideline-Book.pdf

[25] DGFP Digital Archive. Directorate General of Family Planning (DGFP); [cited 2022 Feb 4]. Available from: http://archive.dgfp.gov.bd/

[26] Training Manual on adolescent friendly health services. MCH Services Unit, Directorate General of Family Planning, Ministry of Health and Family Welfare; 2019 [cited 2024 Feb 12]. Available from: http://archive.dgfp.gov.bd/

[27] Ogu R, Maduka O, Alamina F, Adebiyi O, Agala V, Eke G, et al. Mainstreaming youth-friendly health services into existing primary health care facilities: Experiences from South-South Nigeria. International Journal of Adolescent Medicine and Health. 2018 Jan 25;32(3). doi: 10.1515/ijamh-2017-0151 29369812

[28] Napit K, Shrestha KB, Magar SA, Paudel R, Thapa B, Dhakal BR, et al. Factors associated with utilization of adolescent-friendly services in Bhaktapur District, Nepal. Journal of Health, Population and Nutrition. 2020 Feb 10;39(1). doi: 10.1186/s41043-020-0212-2 32041664 PMC7011236

[29] Kundu S, Kundu S, Rahman MdA, Kabir H, Al Banna MdH, Basu S, et al. Prevalence and determinants of contraceptive method use among Bangladeshi women of reproductive age: A Multilevel Multinomial analysis. BMC Public Health. 2022 Dec 16;22(1). doi: 10.1186/s12889-022-14857-4 36526989 PMC9756620

[30] Humayun Kabir., Nirod Chandra, Saha Andrea, L., Wirtz Rukhsana, Gazi. Treatment-seeking for selected reproductive health problems: behaviours of unmarried female adolescents in two low-performing areas of Bangladesh. Reproductive Health, (2014). doi: 10.1186/1742-4755-11-54 25034541 PMC4110240

[31] Li Z, Patton G, Sabet F, Zhou Z, Subramanian SV, Lu C. Contraceptive use in adolescent girls and adult women in low- and middle-income countries. JAMA Network Open. 2020 Feb 19;3(2). doi: 10.1001/jamanetworkopen.2019.21437 32074290 PMC12634135

[32] Vincent Morelli., Chenai Nettey. Adolescent Health Screening: Toward A More Holistic Approach. (2019).1–5. doi: 10.1016/B978-0-323-66130-0.00001–6

[33] Lawrence RS, Sim LJ, Gootman JA, editors. Adolescent health services: Missing opportunities. Washington, DC: National Academies Press; 2009. 25009892

