# Supplementary material for: Experience in implementing adolescent friendly health services in rural districts of Bangladesh
Source: PLOS Glob Public Health. 2024 Nov 14;4(11):e0003930. doi: 10.1371/journal.pgph.0003930 (PMC11563374; doi:10.1371/journal.pgph.0003930)
Supplement: S1 Text — (DOCX) [file pgph.0003930.s001.docx]

**Experience in implementing adolescent friendly health services in rural districts of Bangladesh**

**Table 1: List of reviewed relevant documents on AFHS**

| **Serial No.** | **Name of the document / report** | **Description of the document** | **Objective of the desk review** | **Key findings** |
| --- | --- | --- | --- | --- |
|  | National Adolescent Health Strategy (NAHS) 2017-2030 (Bangladesh) | A national policy document developed by govt. in partnership with experts, professionals and development partners and endorsed | To identify the national policy status and strategic directions for improving the health of the adolescent population | Policy environment of AFHS |
|  | Operational Plan of DGHS and DGFP | It is part of the National Health, Population and Nutrition program 2017-2022, and under implementation by the government | To identify the key activities for implementing the NAHS which also detailed activities for adolescents | Objectives, responsibilities in line with the goal contained within the strategic plan |
|  | A Baseline Survey of AFHS: Household Survey  July to August 2017 | A survey report which was conducted to determine the program parameters for AFHS in the four districts of Bangladesh: Tangail, Gazipur, Khulna and Jamalpur | To explore status and utilization of adolescent health services before the implementation of AFHS in four study districts | Adolescent health problems, access to and utilization of health services: adolescent boys, girls and their mothers’ perspectives |
|  | A Baseline Survey of AFHS: Health Facility Survey August to September, 2017 | A survey report which was conducted to explore availability and readiness of the health facilities to provide services to the adolescents in same four districts | To explore health services provision for adolescents in government health facilities before AFHS program | Hospital readiness for AFHS in 56 public health facilities of 4 districts (Khulna, Jamalpur, Tangail and Gazipur) |
|  | A Baseline Survey of AFHS: Qualitative Study Report  August to September, 2017 | A qualitative study report which was conducted to identify challenges and scopes of improvement for adolescent health services in Bangladesh | To understand an overview of the AFHS services, its utilization, challenges and scopes of improvement for AFHS | Stakeholders’ perception and recommendations about AFHS regarding its use, challenges and future steps: Service managers and gatekeepers |
|  | Implementation Research (IR) report on AFHS  October 2018 to December 2019 | A study report which was conducted to explore the AFHS status for enhancing the facility-based AFHS within the existing government health system | To identify enabling and hindering factors for AFHS after intervention | Socio-economic barriers and solutions: Health care providers, school authorities, adolescents taking service and their parents |
